# Suvanine Sesterterpenes from a Tropical Sponge *Coscinoderma* sp. Inhibit Isocitrate Lyase in the Glyoxylate Cycle

**DOI:** 10.3390/md12105148

**Published:** 2014-10-10

**Authors:** So-Hyoung Lee, Tae Hyung Won, Heegyu Kim, Chan-Hong Ahn, Jongheon Shin, Ki-Bong Oh

**Affiliations:** 1 Department of Agricultural Biotechnology, College of Agriculture and Life Sciences, Seoul National University, Seoul 151-921, Korea; E-Mails: awhee84@naver.com (S.-H.L.); hqhqeori@naver.com (H.K.); chanhong@snu.ac.kr (C.-H.A.); 2 Natural Products Research Institute, College of Pharmacy, Seoul National University, Seoul 151-742, Korea; E-Mail: wth123@snu.ac.kr

**Keywords:** *Candida albicans*, isocitrate lyase, *Coscinoderma* sp., suvanine sesterterpenes, *ICL* mutants

## Abstract

The glyoxylate cycle is a sequence of anaplerotic reactions catalyzed by the key enzymes isocitrate lyase (ICL) and malate synthase (MLS). Mutants of *Candida albicans* lacking *ICL* are markedly less virulent in mice than the wild-type. Suvanine sesterterpenes (**1**−**9**) isolated from a tropical sponge *Coscinoderma* sp. were evaluated for their inhibitory activities toward recombinant ICL from *C. albicans*. These studies led to the identification of a potent ICL inhibitor, suvanine salt (**2**), which possesses a sodium counterion and displays an inhibitory concentration value (IC_50_) of 6.35 μM. The growth phenotype of *ICL* deletion mutants and semi-quantitative reverse transcription-polymerase chain reaction (RT-PCR) analyses indicated that compound **2** inhibits the *ICL* mRNA expression in *C. albicans* under C_2_-carbon-utilizing conditions. The present data highlight the potential for suvanine sesterterpenes treatment of *C. albicans* infections via inhibition of ICL activity.

## 1. Introduction

The glyoxylate cycle, a modified form of the tricarboxylic acid (TCA) cycle, is well documented in archaea, bacteria, protists, plants, fungi, and nematodes [1]. Discovered initially in microorganisms, this cycle plays a fundamental role in the nutrient-limited environment by providing the means for microorganisms to grow on acetate, ethanol or fatty acids [2]. The cycle function has been confirmed by analyzing mutants of pathogenic microorganisms that lack isocitrate lyase (ICL) and malate synthase (MLS), key enzymes in the glyoxylate cycle [3,4]. The genetic regulation of the glyoxylate cycle during microbial growth on acetate has been investigated, and in the last several years it has become evident that this pathway is important in microbial pathogenesis. The expression of *ICL* is upregulated during infection of macrophages by the pulmonary bacterium *Mycobacterium* *tuberculosis* [5,6]. Infection of rice with *Magnaporthe grisea* leads to the expression of genes involved in the glyoxylate cycle [7]. In addition, *ICL*-deletion mutants of these microorganisms show virulence attenuation.

Research on candidiasis in mice has shown that *Candida* *albicans*, the most serious human pathogenic fungus, requires ICL to be fully virulent [8,9]. The genes of the glyoxylate cycle are strongly induced upon phagocytosis of *C. albicans* by macrophages. The interior environment of the phagolysosome is abundant in carbon sources such as fatty acids or their breakdown products, which allows *C. albicans* to utilize the enzymes of the glyoxylate cycle and permits the use of C_2_ carbon sources. The *C. albicans* mutant strain lacking the glyoxylate cycle enzyme ICL is markedly less virulent in a mouse model of systemic candidiasis and less persistent in internal organs than the wild-type strain [8,9,10]. As this cycle does not operate in humans, the key enzymes of the glyoxylate cycle represent promising targets for the control of fungal infection and the development of antifungal drugs. In previous years, a wide array of works developing potential ICL inhibitors have been reported. Various 3-nitropropionamides, pyruvate-isoniazid analogs, salicylanilide and benzanilide derivatives showed a potential to inhibit *M. tuberculosis* ICL [11,12]. As part of efforts to discover pharmacologically effective ICL inhibitors, many marine-derived natural compounds were isolated and evaluated against *C.* *albicans* and *M. grisea* ICL [13,14].

Several of the sponge-derived sesterterpenes and related pentaprenyl hydroquinones [15], represented by the halisulfates and suvanine, possess sulfate groups and exhibit diverse bioactivities such as cytotoxic, antimicrobial [16] and anti-inflammatory properties [17], as well as inhibitory effects on serine protease [18] and CDC25 phosphatase [19]. In addition, recent biological study has shown that HSP60, a chaperone involved in the inflammatory response, is the main cellular target of suvanine [20]. In the course of searching for secondary metabolites of biological significance from marine organisms, we encountered the sponge *Coscinoderma* sp., collected from Chuuk Island, Micronesia. Chemical investigation of this animal led to the isolation of new compounds, suvanine salts and related derivatives [21]. In this study, we investigated the potential for isolated suvanine sesterterpenes as inhibitors of *C. albicans* ICL.

## 2. Results and Discussion

**Figure 1:**
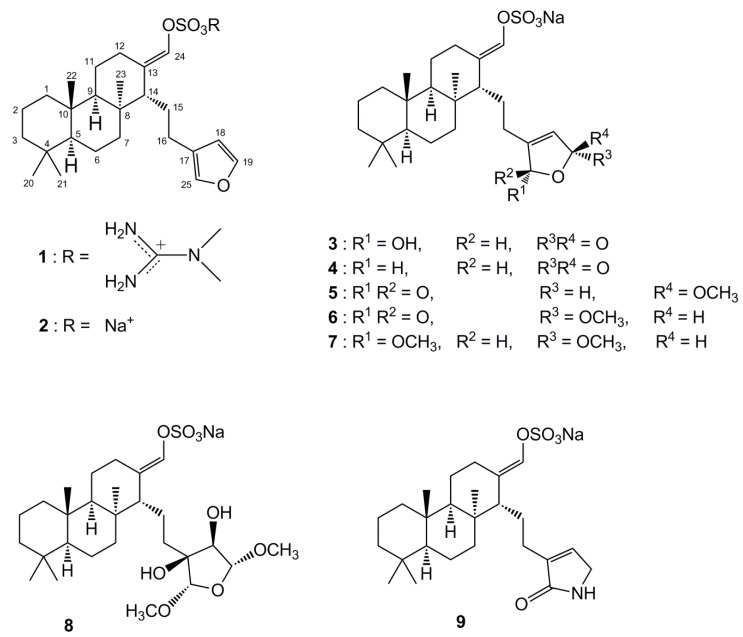
The structures of suvanine sesterterpenes (**1**–**9**).

**Figure 2:**
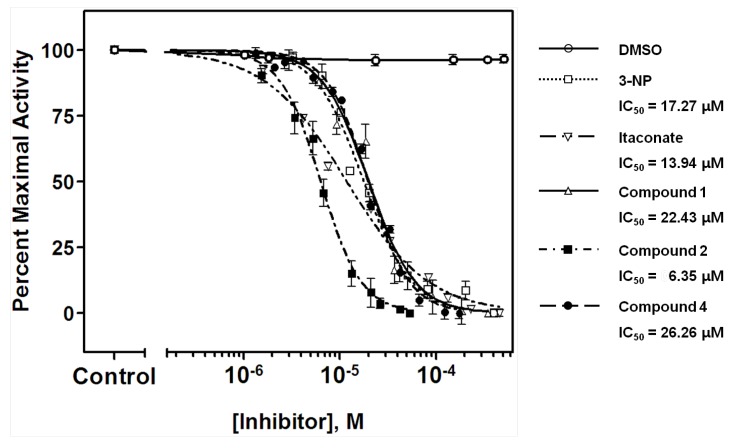
A comparison of the dose–response curves of suvanine sesterterpenes (**1**, **2**, and **4**) against the ICL enzyme from *C.* *albicans* ATCC 10231. Data were scaled to internal controls (0.5% DMSO-treated), and GraphPad ver. 5.0 was used to fit the measured data and determine the IC_50_ values. The results are presented as means ± SD (*n* = 3). 3-Nitropropinate and itaconate were used as the positive controls.

Compound **1**−**9** were obtained as mentioned previously [21] (Figure 1). The expression and purification of recombinant ICL from the genomic DNA of *C. albicans* (ATCC 10231) were carried out by a method described previously [22]. The inhibitory effects of the isolated compounds on ICL were evaluated according to a procedure documented previously [23,24]. The basic concept of this method was to measure spectrophotometrically the formation of glyoxylate phenylhydrazone in the presence of phenylhydrazine and isocitrate. The effect of the inhibitor on ICL was calculated as a percentage relative to dimethyl sulfoxide (DMSO)-treated control. Mixture of ICL, substrate, phenyhydrazine was incubated for 30 min with various concentrations of suvanine sesterterpenes (100 to 0.1 μg/mL). The formation of glyoxylate phenylhydrazone was followed spectrophotometrically at 324 nm. Data were scaled to internal controls, and a four- parameter logistic model (GraphPad ver. 5.0, Prism) was used to fit the measured data and determine IC_50_ (inhibitory concentration for 50% activity) values [25]. The representative dose–response curves of suvanine sesterterpenes (**1**, **2**, and **4**) against the ICL enzyme were compared to that of known ICL inhibitors, 3-nitropropinate and itaconate [12,26] (Figure 2).

The ICL inhibitory potencies (IC_50_) of the isolated compounds **1**−**9** are shown in Table 1. Among the suvanine sesterterpenes, suvanine salts (**1** and **2**) and a butenolide-containing derivative of suvanine (**4**) were found to be strong ICL inhibitors, with IC_50_ values of 22.43, 6.35, and 26.26 μM, respectively. Compound **2** in particular was more effective than 3-nitropropinate (IC_50_ = 17.27 μM) and itaconate (IC_50_ = 13.94 μM). Regarding the suvanine salts, compound **2** with a sodium counterion exhibited much more potent inhibition than **1**, which possessed an *N*,*N*-dimethylguanidium counterion [18]. One possible explanation for the lower potency of compound **1** is that, not only the distance, the spatial orientation of the enolsulfate group relative to that of the furan moiety also plays an important role in ICL inhibitory activity. Compounds **3**–**9** possessed modified furan moieties with varying degrees of oxidation and exhibited lower inhibitory activities than compound **2**. By comparing the chemical structures of the isolated compounds, we found that the ICL inhibitory activities of compounds **3**–**9** were affected markedly by the degree of oxidation of the furan ring (Figure 1). Overall, these results provided important insight regarding the structure−activity relationships of suvanine sesterterpenes.

**Table 1:** Inhibitory activity of compounds **1**–**9** against ICL from *C. albicans* ATCC 10231 and growth of *C. albicans* SC5314.

Compound	ICL IC_50_, µM (µg/mL)	MIC (µg/mL)	
		Glucose	Acetate
**1**	22.43 ± 1.49 (12.03 ± 0.80)	100	25
**2**	6.35 ± 1.37 (3.00 ± 0.65)	100	12.5
**3**	56.19 ± 7.10 (28.33 ± 3.58)	>100	>100
**4**	26.26 ± 3.69 (12.82 ± 1.80)	>100	100
**5**	50.23 ± 6.27 (26.03 ± 3.25)	>100	>100
**6**	96.15 ± 1.54 (49.83 ± 0.80)	>100	100
**7**	59.10 ± 3.45 (27.30 ± 1.85)	>100	>100
**8**	67.64 ± 4.88 (38.44 ± 2.77)	>100	>100
**9**	59.15 ± 1.11 (28.82 ± 0.54)	>100	>100
3-NP	17.27 ± 1.04 (2.06 ± 0.12)	>100	>100
Itaconate	13.94 ± 0.64 (1.66 ± 0.08)	>100	>100
Amph B	ND	1.56	0.39

3-Nitropropinate (3-NP) and itaconate were used as reference inhibitors of ICL; Amphotericin B (Amph B) was used as a standard antifungal drug; 0.5% DMSO was used as a negative control; ND, not determined.

**Figure 3:**
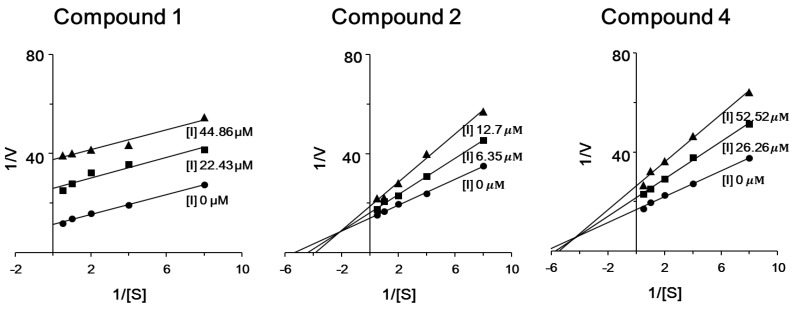
Lineweaver-Burk plot of ICL inhibition by compounds **1**, **2** and **4**. [S], substrate concentration [mM]; *V*, reaction velocity (∆absorbance unit/min). Each data point represents the mean of three experiments.

To determine the type of inhibition, kinetic studies were performed with compounds **1**, **2** and **4** at IC_50_ or twofold IC_50_ based on Lineweaver and Burk plot [27] (Figure 3). Inhibitor constants were obtained by Dixon plot. Inhibitory kinetics show that compound **1** behaved as an uncompetitive inhibitor (*K*_i_ = 17.38 μM). Compound **2** (*K*_i_ = 14.43 μM) and **4** (*K*_i_ = 62.57 μM) behaved as mixed noncompetitive inhibitors. Moreover, the binding of compounds **1**, **2** and **4** to enzyme was reversible because the enzyme activity was indeed recovered by dialysis within 2 h, excluding the possible existence of a covalent bond between inhibitor and enzyme.

The microbial strategy for survival during growth in a nutrient-free environment entails a metabolic shift in the carbon source to C_2_ substrates generated by β-oxidation of fatty acids [1,2]. Under these conditions, glycolysis is decreased and the glyoxylate shunt is upregulated significantly to allow anaplerotic maintenance of the TCA cycle and assimilation of carbon via gluconeogenesis. To investigate whether ICL inhibitors affected C_2_ substrate use, we cultured *C. albicans* strain SC5314 in YNB broth containing either 2% glucose or 2% potassium acetate as the sole carbon source, and inhibition was evaluated based on the MIC. *C. albicans* strain SC5314 was used in this test as the ICL sequence alignment of this strain (GenBank accession number AF222905) showed 100% identity with that of *C. albicans* ATCC 10231 over its entire length (data not shown). As shown in Table 1, fungal growth inhibition tests indicated that suvanine sesterterpenes **1**–**9** at concentrations up to 100 μg/mL had weak to no inhibitory effects on SC5314 cultured in glucose. Compounds **1** and **2** at concentration 100 μg/mL showed antifungal activities on SC5314 cultured in glucose. Therefore, at high concentrations, it is possible that these compounds could affect other molecular targets in *C. albicans* cells [16,17,18,19,20]. A similar trend was observed in SC5314 cultured in acetate with all compounds, except for compounds **1** and **2**. Compound **2**, which showed particularly potent inhibitory activity against SC5314 cultured in acetate, with an MIC value of 12.5 μg/mL. The antifungal activity of compounds **1**–**9** was also evaluated against *C. albicans* strains ATCC 11006 (from human virginal tracts), ATCC 10261 and ATCC 18804 (from human skin lesin). As expected, these compounds at concentrations up to 100 μg/mL had no inhibitory effects on *C. albicans* strains cultured in glucose, but were inhibitory to these strains cultured in acetate. These results indicated that ICL is involved in the proliferation of *C. albicans* using C_2_ substrates.

**Figure 4:**
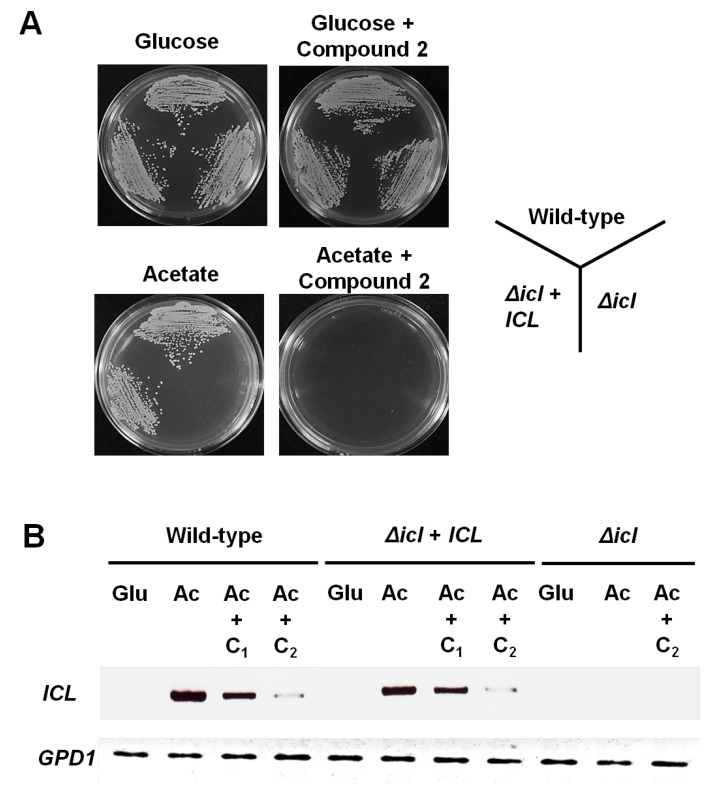
Inhibitory activity of compound **2** against the growth and *ICL* mRNA expression of the wild-type and *Δicl* mutants. (**A**) *C. albicans* strain SC5314 (wild-type), MRC10 (Δ*icl*), and MRC11 (Δ*icl* + *ICL*) were cultured on YNB agar plates containing the indicated carbon source (2% glucose or 2% sodium acetate) with or without compound **2** (12.5 µg/mL) for 3 days at 28 °C; (**B**) Strains were grown to mid-log phase in minimal YNB liquid medium containing 2% glucose. Cells were collected by centrifugation and shifted to the same medium containing 2% glucose (Glu), 2% sodium acetate (Ac), or 2% sodium acetate (Ac) plus compound **2** (C1, 6.25 µg/mL; C2, 12.5 µg/mL) for 4 h at 28 °C. Total RNA was prepared from these cells and *ICL* mRNA expression was determined by semi-quantitative RT-PCR analysis. The *GPD1* housekeeping gene was used as a loading control.

The genes encoding the glyoxylate cycle are required for virulence in both *M. tuberculosis* and *C. albicans* that can survive within macrophages [5,8]. It was expected that inhibitors of the glyoxylate cycle pathway would block the nutrient availability and prevent survival of these pathogens inside the macrophage. Based on these findings, we next investigated the effects of compound **2** on the growth phenotype and *ICL* mRNA expression of *C. albicans* strain SC5314 (wild-type), MRC10 (Δ*icl*), and MRC11 (Δ*icl* + *ICL*) [9]. As shown in Figure 4A, these strains grew normally on YNB agar plates containing glucose or glucose plus 12.5 μg/mL compound **2**. However, the *ICL*-deletion mutant MRC10 failed to grow on acetate as the sole carbon source. In addition, the growth of all tested strains was inhibited markedly on YNB agar plates containing acetate plus 12.5 μg/mL compound **2**. The effects of compound **2** on *ICL* mRNA expression in *C. albicans* were investigated using semi-quantitative RT-PCR analysis. As shown in Figure 4B, the *ICL* transcript levels in SC5314 and MRC11 were undetectable when the YNB liquid medium contained glucose, but were strongly induced when the medium contained acetate. Interestingly, under the *ICL* expression conditions, the transcript levels of *ICL* in both strains were diminished with increasing compound **2** concentrations. At 12.5 μg/mL, compound **2** reduced *ICL* mRNA expression by seventeenfold. These findings indicated that compound **2** inhibits *ICL* expression in *C. albicans* under C_2_-carbon-utilizing conditions.

In this study, however, we investigated the effect of suvanine salt on the *ICL* mRNA expression in *C. albicans*. Further studies are required to identify the relationship between enzyme inhibition and inhibition of gene expression, and the main cellular target of this compound. In previous works, suvanine sesterterpenes functionally inhibited enzyme activity such as serine protease [18], CDC25 phosphatase [19]. We also found that these sesterterpenes (**1**–**9**) have potent Na^+^/K^+^-ATPase inhibitory activities and these activities were influenced by structure bearing sulfonate group [21]. There is no direct evidence to identify how sulfonate of compounds inhibits Na^+^/K^+^-ATPase. However, strong supposition is that sulfonate containing compound modifies a specific conformationally sensitive amino residue on Na^+^/K^+^-ATPase, resulting in loss of enzyme activity [28]. Sulfur-containing natural products have unique chemical and biochemical properties linked to redox process, metal binding and catalytic reactions [29]. Therefore, suvanine sesterterpenes are expected to have various bioactivities.

## 3. Experimental Section

### 3.1. General Experimental Procedure 

Optical rotations were measured on a JASCO P-1020 polarimeter (JASCO Co., Tokyo, Japan) using a 1-cm cell. Ultraviolet (UV) spectra were recorded on a Hitachi U-3010 spectrophotometer (Hitachi High-Technologies Co., Tokyo, Japan). Infrared (IR) spectra were recorded on a JASCO 300E FT-IR spectrometer (JASCO Co., Tokyo, Japan). Bruker Avance 600 MHz spectrometer (Bruker, Rheinstetten, Germany) was used to obtain ^1^H, ^13^C and two-dimensional nuclear magnetic resonance (NMR) spectra. Mass spectrometric data were obtained at the Korea Basic Science Institute (Daegu, Korea) and were acquired using a JEOL JMS 700 mass spectrometer (JEOL Ltd., Tokyo, Japan) using meta-nitrobenzyl alcohol as a matrix for the fast atom bombardment mass spectrometry (FABMS). High-performance liquid chromatography (HPLC) was performed using a Spectrasystem p2000 (Thermo Scientific, Waltham, MA, USA) equipped with a refractive index detector, Spectrasystem RI-150. All solvents were spectroscopic grade or distilled from glass prior to use.

### 3.2. C. albicans Strains and Growth Media

*C. albicans* strain ATCC 10231 was the source of the *ICL* gene. *C. albicans* strains used for growth assay were SC5314 (ATCC MYA-2876) (wild-type), MRC10 (Δ*icl*), MRC11 (Δ*icl* +*ICL*), ATCC 10261, ATCC 18804, and ATCC 11006. The *ICL*-deletion mutant strains MRC10 and MRC11 were kindly provided by Prof. Michael C. Lorenz (The University of Texas Health Science Center at Houston, USA) [9]. These strains were subcultured in yeast nitrogen base (YNB) broth (Difco Laboratories, Detroit, MI, USA) containing 2% glucose at 28 °C. Stock solutions of test compounds were prepared by dissolution in DMSO and stored at −20 °C until use. The final concentration of DMSO was 0.5% in all assays, which was found to have no effect on the enzyme activity at a concentration of less than 1%. Other general reagents were obtained from commercial suppliers and were of the highest grade available.

### 3.3. Preparation of Recombinant ICL

The preparation of recombinant ICL protein from the genomic DNA of *C. albicans* ATCC 10231 was carried out using a method described previously [22]. Briefly, *ICL* was PCR-amplified using two synthetic primers: 5′-AGAATTCCTACCATGCCTTACACTCC-3′ (forward) and 5′-CTTCGTCGACTCAAAATTAAGCCTTG-3′ (reverse). The PCR product was cloned into the pBAD/Thio-TOPO vector (Invitrogen, Carlsbad, CA, USA) and transformed into *Escherichia coli* TOP10 (Invitrogen). After arabinose (0.02%) induction of cultures at 25 °C for 8 h, cells were lysed by lysozyme treatment and sonication, and the recombinant protein was purified using a Ni-NTA affinity column (Qiagen, Hilden, Germany) according to the manufacturer’s protocol.

### 3.4. ICL Inhibitor Potency Determination

The effect of isolated compounds on ICL was evaluated according to a procedure documented previously [23,24]. A 1-mL aliquot of the reaction mixture contained 20 mM sodium phosphate buffer (pH 7.0), 1.27 mM *threo*-dl (+) isocitrate, 3.75 mM MgCl_2_, 4.1 mM phenylhydrazine and 2.5 μg/mL purified ICL. The reaction was performed at 37 °C for 30 min with and without a prescribed concentration of the inhibitor dissolved in DMSO (final concentration, 0.5%). The formation of glyoxylate phenylhydrazone was followed spectrophotometrically at 324 nm. The effect of the inhibitor on ICL was calculated as a percentage relative to the solvent-treated control. A 12-point, twofold serial dilution dose–response assay was performed in triplicate. The resultant dose–response concentration range was 100 to 0.1 μg/mL of inhibitor in a 1-mL final reaction volume. Data were scaled to internal controls, and a four-parameter logistic model (GraphPad ver. 5.0, Prism) was used to fit the measured data and determine the IC_50_ values [25]. The ICL inhibitor 3-nitropropionate was used as a positive control [26]. Protein concentration was determined by the method of Bradford [30] using the Bio-Rad protein assay kit (Bio-Rad, Hercules, CA, USA) and bovine serum albumin as a standard.

### 3.5. In Vitro Growth Assays

*C. albicans* strains were grown in YNB (6.7% yeast nitrogen base) broth containing 2% glucose at 28 °C for 24 h, harvested by centrifugation, and washed twice with sterile distilled water. Each test compound was dissolved in DMSO and diluted with YNB containing 2% glucose or 2% potassium acetate to prepare serial twofold dilutions in the range 100–0.1 μg/mL. The final DMSO concentration was maintained at 0.5% by adding DMSO to the medium. Twenty microliters of the broth containing test fungus was added to each well of a 96-well microtiter plate (final concentration of 1 × 10^4^ cells/mL). Culture plates were incubated for 3 days at 28 °C. The minimum inhibitory concentration (MIC) values were determined to be the lowest concentration at which the test compounds inhibited fungal growth. Amphotericin B was used as a reference compound.

### 3.6. Semi-Quantitative RT-PCR Analysis

*C. albicans* strain SC5314 (wild-type), MRC10 (Δ*icl*), and MRC11 (Δ*icl* +*ICL*) were grown into the mid-log phase in YNB broth (2% glucose), collected by centrifugation and washed twice with sterile distilled water. Cells were resuspended in YNB media containing 2% glucose, 2% acetate, or 2% acetate plus compound **2** (6.25 and 12.5 µg/mL) and grown for 4 h at 28 °C. Total RNA from each sample was isolated by using RNeasy Mini Kit (Qiagen), and 1 μg of total RNA was reverse transcribed into cDNA using the Superscript III First-Strand Synthesis System (Invitrogen). Semi-quantitative RT-PCR was conducted using the *ICL*-specific primers: 5′-ATGCCTTACACTCCTATTGACATTCAAAA-3′ (forward) and 5′-TAGATTCAGCTTCAGCCATCAAAGC-3′ (reverse). The *GPD1* (glycerol-3-phosphate dehydrogenase) housekeeping gene was used as a loading control with the specific primers: 5′-AGTATGTGGAGCTTTACTGGGA-3′ (forward) and 5′-CAGAAACACCAGCAACATCTTC-3′ (reverse). PCR amplification was started with an initial incubation at 98 °C for 10 min followed by 30 cycles of 30 s at 98 °C for, 30 s at 56 °C, and 30 s at 72 °C and then performed at 72 °C for 5 min. The *ICL* mRNA expression level was determined by densitometric analysis using the ImageJ software (NIH, Bethesda, MD, USA).

## 4. Conclusions

Suvanine sesterterpenes (**1**–**9**) were isolated from the sponge *Coscinoderma* sp., and their inhibitory activities were investigated against ICL from *C. albicans*. These studies led to the identification of compounds **1**, **2**, and **4** as potent inhibitors. Compound **2** in particular, which possesses a sodium counterion, was most effective. The preliminary structure–activity relationship study suggested that the ICL inhibitory activities of suvanine sesterterpenes are affected markedly by the distance and spatial orientation of the enolsulfate group relative to that of the furan ring. This analysis provides potentially useful information on advanced drug design strategies to identify novel ICL inhibitors. The growth phenotype of *ICL* deletion mutants and semi-quantitative RT-PCR analysis indicated that compound **2** inhibited *ICL* mRNA expression in *C. albicans* under C_2_-carbon-utilizing conditions. As the enzymes of the glyoxylate cycle are not found in mammals, this compound shows promise as an antifungal agent in terms of suppressing *C. albicans* virulence. Further *in vivo* studies on this compound are underway in our laboratory.
